# On the Firing Rate Dependency of the Phase Response Curve of Rat Purkinje Neurons *In Vitro*

**DOI:** 10.1371/journal.pcbi.1004112

**Published:** 2015-03-16

**Authors:** João Couto, Daniele Linaro, E De Schutter, Michele Giugliano

**Affiliations:** 1 Theoretical Neurobiology and Neuroengineering Laboratory, University of Antwerp, Antwerpen, Belgium; 2 NeuroElectronics Research Flanders, Leuven, Belgium; 3 Computational Neuroscience Unit, Okinawa Institute of Science and Technology Graduate University, Onna, Okinawa, Japan; 4 Department of Computer Science, University of Sheffield, Sheffield, United Kingdom; 5 Brain Mind Institute, EPFL, Lausanne, Switzerland; École Normale Supérieure, College de France, CNRS, FRANCE

## Abstract

Synchronous spiking during cerebellar tasks has been observed across Purkinje cells: however, little is known about the intrinsic cellular mechanisms responsible for its initiation, cessation and stability. The Phase Response Curve (PRC), a simple input-output characterization of single cells, can provide insights into individual and collective properties of neurons and networks, by quantifying the impact of an infinitesimal depolarizing current pulse on the time of occurrence of subsequent action potentials, while a neuron is firing tonically. Recently, the PRC theory applied to cerebellar Purkinje cells revealed that these behave as phase-independent integrators at low firing rates, and switch to a phase-dependent mode at high rates. Given the implications for computation and information processing in the cerebellum and the possible role of synchrony in the communication with its post-synaptic targets, we further explored the firing rate dependency of the PRC in Purkinje cells. We isolated key factors for the experimental estimation of the PRC and developed a closed-loop approach to reliably compute the PRC across diverse firing rates in the same cell. Our results show unambiguously that the PRC of individual Purkinje cells is firing rate dependent and that it smoothly transitions from phase independent integrator to a phase dependent mode. Using computational models we show that neither channel noise nor a realistic cell morphology are responsible for the rate dependent shift in the phase response curve.

## Author Summary

The phase response curve (PRC) quantifies the effect of an infinitesimal perturbation on the phase of an oscillator, be it mechanical, electronic or biological. In the particular case of neurons, PRCs can be employed to infer several network properties that are influenced by intrinsic membrane mechanisms. It has been shown that the PRC of tonically firing Purkinje Cells is flat at low firing rates, which has profound implications for information processing in the cerebellum. Here, we propose a novel method to estimate the PRC of single Purkinje cells at various firing rates and use it to unveil the smooth transition between flat and phasic PRC. Furthermore, we address potential explanations for the observed transition using computational modeling.

## Introduction

The intrinsic electrical activity of Purkinje cells (PCs) exhibits a large repertoire of dynamical behaviors, including spontaneous firing of simple action potentials (APs), bistability of the firing rate, and hysteresis [1–4]. In addition, the extended range of PCs firing rates during behavior suggests that the rate of APs, its sudden transitions, its coherence across PCs, and the AP timing synchronization may contribute to information representation, processing, and downstream relaying. Thus, investigating how distinct firing regimes affect spontaneous and evoked response properties is imperative for dissecting cerebellar computation. Recently, key results from the mathematical theory of coupled oscillators sparked a lot of interest: a simple input-output characterization of the units composing a network, known as their *phase response* (or *phase resetting*) *curve* (PRC), is sufficient to classify and predict individual and collective properties. In the context of tonically firing neurons, the PRC quantifies the impact of an infinitesimal depolarizing current pulse on the time of occurrence of subsequent APs [5–10]. As the cell oscillates regularly, the pulse advances or delays the time of the next AP, depending on the oscillation *phase* *φ* corresponding to the time of pulse delivery. The resulting change of the time of the next AP can also be quantified in terms of the cell’s firing period and thus expressed as a *phase shift* Δ*φ*. By capturing the relationship between the evoked *phase shift* Δ*φ* and the *phase* *φ* at which the input pulse occurred, the PRC predicts how, upon receiving weak synaptic inputs, neurons transiently delay or accelerate AP firing, contribute to network-wide AP synchrony, integrate external inputs or detect their temporal coincidences. So far, not only has the PRC been considered in theoretical and computational studies, but it has also been computed in experimental works (see [11] for a review), where different methods have been devised for its estimation [11–13]. Recently, Phoka et al. 2010 [14] proposed a correction to a traditional estimation method and tested it in PCs of juvenile mice. Unexpectedly, they reported that the PC’s intrinsic firing rate has a profound effect on the response properties: the PRC of PCs firing at low rates displays a flat profile, suggesting that neurons behave like phase-independent inputs integrators; on the other hand, the PRC of PCs firing at high firing rates has a prominent peak, indicating a phase preference similar to coincidence detectors. While it was not the first time that PRCs were shown to undergo changes over a range of AP frequencies [15], the wide physiological range of PCs spontaneous firing rates and their ease of experimental access in *in vitro* preparations, made the report on the rate dependence of the PRC relevant. Furthermore, intrinsic membrane properties might promote synchrony in a way that is relevant to information processing [16], particularly in the cerebellum [17, 18].

Inspired by these perspectives, here we focused on revisiting, improving, and extending the earlier experimental characterization of Purkinje cells’ PRCs. We aimed at its systematic exploration, both at the single-cell and at the population levels, which may be directly relevant for modeling studies. In particular, in the light of the known bistable behavior of PCs, and their ability to abruptly toggle between distinct AP firing rates, we found it urgent to clarify whether the changes in PRC occur abruptly or smoothly, for increasing AP frequencies. In addition, we tested the effect of the current pulse amplitude, verifying that the PRC prominent phase-dependency of PCs firing at high rates is an intrinsic property and not an artifact of the stimulation protocol. The key contribution of this work is twofold: (i) we developed a novel *ad hoc* closed-loop electrophysiological protocol to regulate PCs slow scale adaptation and achieve highly significant PRC estimates at fixed firing rates in a relatively short experimental time. By such an approach, (ii) we confirmed and extended the observations of [14], considerably improving the earlier observation statistics, and demonstrating conclusively and unambiguously that no abrupt switch in PRC occurs. Instead, PCs smoothly shift from integrators to coincidence detectors, as their AP frequency increases. Finally, verifying that these observations are not affected by the particular PRC estimation method, we tested our conclusions employing both the corrected direct method, as in [14], and an indirect method [11–13].

## Materials and Methods

### Ethics statement

All procedures were performed according to institutional and national ethical guidelines (license no. LA1100469 from the Belgian Federal Public Service Health, Food Chain Safety and Environment).

### Acute brain tissue slices preparation

Cerebellar acute slices (sagittal, 250 μm thick) were prepared from 15- to 25-days-old Wistar rats, employing 4% isoflurane anesthesia and rapid decapitation, as described in [19]. Briefly, after isolating the cerebellar vermis, the tissue was glued with cyanoacrylate glue to a flat metal platform and surrounded by agar blocks to improve stability during slicing [3, 20]; the tissue was then cut in 250 μm slices using a vibratome (VT1000S, Leica Microsystems, Wetzlar, Germany) in ice-cold artificial cerebrospinal fluid (ACSF), containing (in mM): 125 NaCl, 2.5 KCl, 1.25 NaH_2_PO_4_, 26 NaHCO_3_, 25 glucose, 2 CaCl_2_, and 1 MgCl_2_, balanced with 95% O_2_ and 5% CO_2_. The slices were incubated for 30 – 45 min at 32°C and then stored at room temperature, until they were transferred to the recording chamber of a fixed-stage upright microscope (DMLFS, Leica Microsystems, Wetzlar, Germany). The microscope was equipped with differential interference contrast (DIC) video-microscopy and mounted a 63x water immersion objective.

### Whole-cell patch clamp electrophysiology and pharmacology

Purkinje cells (PCs) were visually targeted for somatic patch-clamp recordings, upon visual identification by size and location within the cerebellar microcircuitry, under DIC. Some PCs were filled with Lucifer yellow and imaged by epifluorescence microscopy, confirming that the entire dendrite was always in the plane of the slice. Whole-cell patch-clamp recordings were performed at 33 ± 1°C, employing an EPC10 amplifier (HEKA, Lambrecht/Pfalz, Germany) or an Axon Multiclamp 700B amplifier (Molecular Devices, USA), both used in current-clamp mode. Patch electrodes were pulled from thick-walled borosilicate glass capillaries (1BF150, World Precision Instruments, Hitchin, UK) with a horizontal puller (P97, Sutter, Novato, USA) to a resistance of 3 – 6 MΩ. Electrodes were filled with an intracellular solution containing (in mM): 130 methanesulfonic acid, 10 HEPES, 7 KCl, 0.05 EGTA, 2 Na_2_ATP, 2 MgATP, 0.5 Na_2_GTP, and pH adjusted to 7.3 with KOH. All recordings were obtained employing ACSF as the extracellular solution, balanced with 95% O_2_ and 5% CO_2_, and routinely supplemented with 10 μM SR95331 (a selective antagonist of GABA_A_ receptors) to abolish incoming spontaneous synaptic potentials.

Amplified analog signals were low-pass filtered at 10 kHz, sampled at a rate of 30 kHz and digitized at 16 bits with a DAQ board (PCI-6229, National Instruments, USA). The same board was used to generate the amplifier control commands waveforms, synthesized at the same rate and resolution of the data acquisition. Stimulation and response data were generated and collected by using the public domain software LCG [21], and analyzed by custom scripts written in MATLAB (The Mathworks, Natick, MA). The amplifier built-in on-line capacitance compensation circuitry was always applied, while the on-line bridge balancing circuitry was employed alternatively to the off-line (software) active electrode compensation (AEC) [22], whose implementation is built-in in LCG [21]. Liquid junction potentials were left uncorrected and all the chemicals and drugs were obtained from Sigma-Aldrich (Diegem, Belgium). Analysis scripts, LCG configuration files and LCG command line strings to precisely replicate our experimental protocol and modeling are available from ModelDB [23] at http://senselab.med.yale.edu/modeldb (accession number 155735).

### Phase response curve estimates

**Fig 1:**
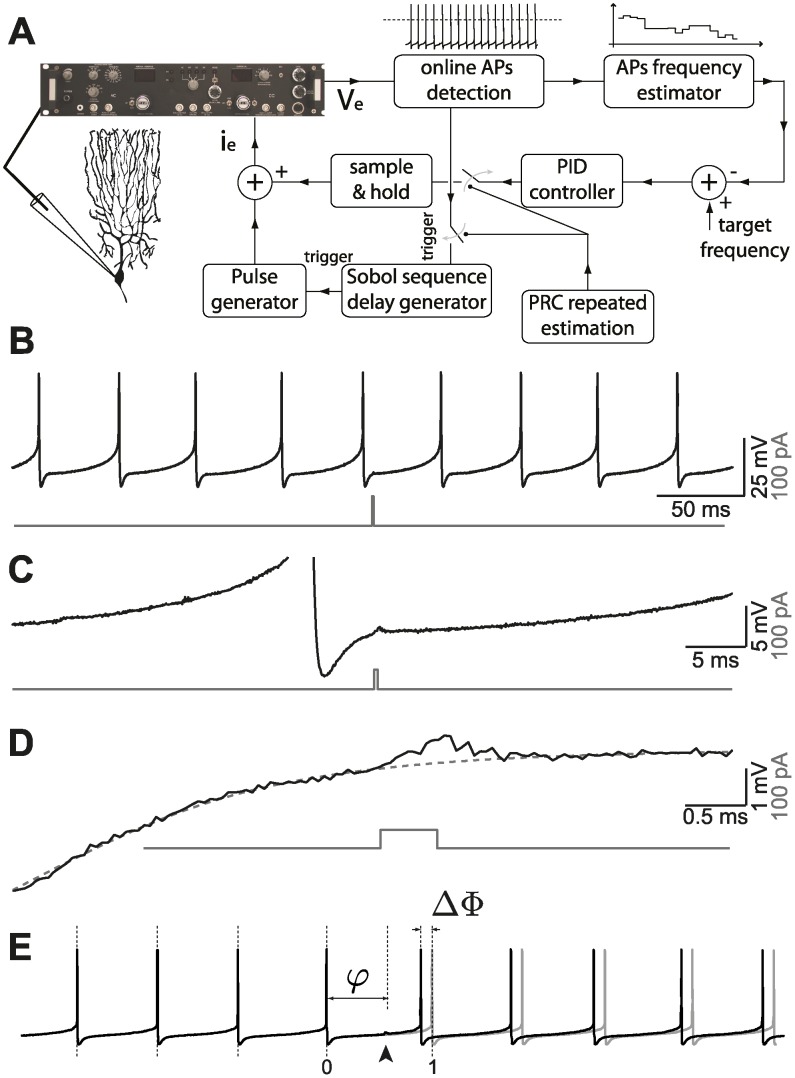
Experimental set-up. (A) A real-time closed-loop in vitro set-up for experimentally estimating the PRC at a fixed firing rate, based on a Proportional-Integral-Derivative (PID) controller and on a reactive-clamp paradigm, was employed to rapidly and optimally explore the dependency of the PRC of PCs on the cell’s firing rate. To this aim, a brief external current-pulse (B-D at different scales) was repeatedly delivered at different phases *φ* of the cell cycle (E), and the resulting impact on the time of the next AP was quantified as a phase delay or of advance Δ*φ* (E).

PRCs were experimentally estimated using direct and indirect methods [11, 13]. Applying direct methods [24] in tonically firing cells, such as the PCs, required the repeated injection of very brief square pulses of current (i.e., *I*_*pulse*_ = 50 − 150 pA, *T*_*pulse*_ = 0.5 − 1 ms, at least 1400 repetitions), each timed at a different phases *φ* of the cell firing cycle (e.g., Fig. 1, panels E and A). The phase-shift Δ*φ* of the next AP induced by each pulse (Fig. 1, panels E, B and C), was first quantified and then normalized by the total injected charge *Q* = *I*_*pulse*_ ⋅ *T*_*pulse*_, [8, 25] allowing comparison across stimulation conditions. Briefly, upon (online or offline, see below) digital detection of the timing *t*_*k*_ of individual AP peaks, the mean ⟨*ISI*⟩ of the distribution of inter-spike intervals *ISI*_*k*_ = (*t*_*k*+1_ − *t*_*k*_) was computed, and taken as an estimate of the (regular) firing period. The occurrence of each pulse was expressed as the corresponding phase *φ* = *τ*/⟨*ISI*⟩, by relating its absolute time of occurrence *t*_*pulse*_ to the AP immediately before (i.e., say *t*_*j*_), *τ* = *t*_*pulse*_ − *t*_*j*_. Note that due to jitter in the next AP, the value of *φ* may slightly exceed its upper theoretical limit *φ* = 1 (i.e., *φ* ∈ [0; 1 + *ε*]). Because with no pulse the next AP would have occurred at *t*_*j*_ + ⟨*ISI*⟩, the actual phase-shift induced by the external perturbation was determined as Δ*φ* = (⟨*ISI*⟩ − *ISI*_*perturbed*_)/⟨*ISI*⟩, with *ISI*_*perturbed*_ = *t*_*j*+1_ − *t*_*j*_ and where *t*_*j*+1_ is the (perturbed) time of the AP immediately following the pulse. By this convention, positive (negative) values of Δ*φ* represent phase advances (delays). Finally, normalizing Δ*φ* to the charge *Q* of each pulse, the traditional direct estimate of the PRC can be expressed as:

$$\begin{array}{c} Z\left(\varphi \right)=\frac{\left\langle ISI\right\rangle -{ISI}_{perturbed}}{\langle ISI\rangle \cdot Q}. \end{array}$$ (1)

However, although *φ* ∈ [0; 1 + *ε*], *Z*(*φ*) cannot be sampled homogeneously by definition. In fact, since *t*_(*j*+1)_ cannot precede *t*_*pulse*_, an upper bound always limits *Z*(*φ*) (i.e., *ISI*_*perturbed*_ ≥ *φ* ⋅ ⟨*ISI*⟩, thus *Z*(*φ*) ≤ (1 − *φ*)/*Q*). We therefore considered an unbiased and more accurate direct method, employing the correction proposed in [14]. This method uses information from higher-orders PRCs [11], including the contributions from the two APs preceding the pulse: *t*_*pulse*_ was also related to the time of the *second* preceding AP (i.e., *t*_*j*−1_), *τ*_2_ = *t*_*pulse*_ − *t*_*j*−1_ and expressed as *τ*_2_ = (*τ* + *ISI*_(*perturbed*−1)_)/⟨*ISI*⟩, with *ISI*_(*perturbed* − 1)_ = *t*_*j*_ − *t*_(*j*−1)_. Note that due to jitter in the APs before and after the pulse, *φ*_2_ may slightly exceed its theoretical limits (i.e., *φ*_2_ ∈ [1 − *ε*; 2 + *ε*]) and thus sample part of the domain of *Z*(*φ*). The phase-shift of the AP preceding the perturbation can be expressed as Δ*φ*_2_ = (⟨*ISI*⟩ − *ISI*_*perturbed*−1_)/⟨*ISI*⟩ and thus the higher order PRC can be written as

$$\begin{array}{c} Z_{2}\left(\varphi_{2}\right)=\frac{\left\langle ISI\right\rangle -ISI_{perturbed-1}}{\langle ISI\rangle -Q}. \end{array}$$ (2)

It can be proven that *Z*_2_(*φ*_2_) ≤ (2 − *φ*_2_)/*Q* and that *Z*_2_(*φ*_2_) ≥ (1 − *φ*_2_)/*Q*: thanks to the AP jitter, *Z*_2_(*φ*_2_) can restore an unbiased estimate of the domain of *Z*(*φ*), precisely above its upper bound, where *Z*(*φ*) could not be properly determined. The unbiased direct estimate of the PRC was then obtained by joining the data sets *Z*(*φ*) ∪ *Z*_2_(*φ*_2_); with *φ*, *φ*_2_ ∈ [0; 1] and indicated for simplicity as *Z*(*φ*) in the following.

Concerning the indirect methods for the PRC estimate, we employed the Weighted Spike-Triggered Average (WSTA), reviewed in [13]. Despite its potential bias due to the non-stationary firing regimes, we used it here solely as a control method and for confirming the firing rate dependency of the PRC in PCs. In tonically firing cells, such as the PCs, WSTA required repeatedly recording the times {*t*}_*k*_ of APs elicited by weak-amplitude fluctuating currents *I*(*t*), generated as exponentially filtered white-noise [26] *ξ*(*t*), lasting for ∼ 30 s:

$$\begin{array}{c} \tau_{I}\cdot \dot{I}\left(t\right)=-I\left(t\right)+s\sqrt{2\cdot \tau_{I}}\cdot \xi \left(t\right), \end{array}$$ (3)

where the steady-state variance *s* ^2^ and autocorrelation time-constant *τ*_*I*_ of the injected current were chosen as 25 – 75 pA and 4 ms, respectively (*τ*_*I*_ ≪ ⟨*ISI*⟩). Stimuli were applied at least twice for each firing rate, employing each time distinct realizations of *I*(*t*) and additional offsets to induce distinct discharge frequencies. For each inter-spike interval *ISI*_*k*_ = (*t*_*k*+1_ − *t*_*k*_), the corresponding portion of *I*(*t*) was isolated and rescaled to the same duration, *I*_*k*_(*φ*) = *I*(*φ*); *φ* = *t*/*ISI*_*k*_, *t* ∈ [*t*_*k*_; *t*_*k*+1_]. The PRC was approximated by the sum of the portions *I*_*k*_(*φ*), weighted by *α*_*k*_ = ⟨*ISI*⟩/*ISI*_*k*_ − 1 and after normalization by the area of the autocorrelation function of *I*(*t*) [27]:

$$\begin{array}{c} Z\left(\varphi \right)≃\frac{\sum_{k}\alpha_{k}\cdot I_{k}\left(\varphi \right)}{2\cdot s^{2}\cdot \tau_{I}}. \end{array}$$ (4)

### PRC data smoothing for the direct method

Instead of binning and constructing a histogram of the sampled PRC data-points, a standard nonparametric smoothing technique based on Gaussian kernel convolution [28] was employed as in [14], aimed at increasing the signal-to-noise ratio of each PRC estimate:

$$\begin{array}{c} \begin{array}{cc} \tilde{Z}\left(\varphi \right)=\frac{\int_{0}^{1}K(\varphi -x)\cdot Z(x)dx}{\int_{0}^{1}K(x)dx} & K\left(x\right)=\frac{1}{\sqrt{(2\pi h^{2}}}e^{-\frac{x^{2}}{2h^{2}}} \end{array} \end{array}$$ (5)

In discrete coordinates, the convolution integral became a sum over each of the *N* data points available, with the kernel *K*(*x*) centered over each available phase. The optimal kernel *bandwidth* *h* was directly inferred from the data [28] as $h=\sqrt{h_{\varphi }\cdot h_{Z}}$, where

$$\begin{array}{c} \begin{array}{cc} h_{\varphi }={\left(\frac{4}{3N}\right)}^{\frac{1}{5}}\frac{median\left\{|\varphi -\varphi_{median}|\right\}}{0.6745} & h_{\varphi }={\left(\frac{4}{3N}\right)}^{\frac{1}{5}}\frac{median\left\{|Z\left(\varphi \right)-Z{\left(\varphi \right)}_{median}|\right\}}{0.6745} \end{array} \end{array}$$ (6)

Throughout the text and in the figures, the smoothed PRC estimates have been indicated for simplicity as *Z*(*φ*).

### Closed-loop stimulation to achieve accurate frequency-clamp

Due to the intrinsic variability of each cell’s inter-spike intervals, repeating the pulse injections over and over in time conveniently allowed us to sample uniformly the range of *φ*, while stimulating the cell at a frequency much lower than its firing rate (i.e., 2–6 pulse/sec). As a consequence of the need to study the firing rate dependency, large parts of the recording were often discarded when the cell was not firing at a fixed rate, further increasing the time needed to obtain a PRC estimate (often greater than 30 min for a single firing rate).

Even though PCs *in vitro* fire spontaneously with a range of AP rates, we aimed at studying systematically the rate-dependency of the PRC in the same neuron. In a first series of experiments, a constant holding current was therefore applied (i.e., on the top of *I*_*pulse*_ or of *I*(*t*)), adapting its value manually from −0.2 to 1 nA, to alter the firing rate of PCs by depolarizing or hyperpolarizing their membranes. Depolarizing or hyperpolarizing a cell instantaneously alters its firing rate, although several minutes are typically required for the cell to reach a (new) steady firing rate. This caused long waiting intervals before estimating the PRC at a given firing rate. In addition, occasional slow drifts of the mean inter-spike intervals occurred, over a window of several seconds, thus altering or biasing the shape of the PRC. To address these limitations, in an additional set of experiments, we made use of a spike rate controller [29], using a closed-loop paradigm similar to the one employed in [30] and inspired by the response-clamp paradigm [31] was adopted: the frequency-clamp. In short, an iterative estimate ${\tilde{F}}_{n}$ of the cell’s instantaneous firing rate was updated online after each AP, detected in real-time (i.e., as a positive crossing of a voltage threshold), using the following formula:

$$\begin{array}{c} {\tilde{F}}_{k}={ISI_{k}}^{-1}\cdot \left(1-e^{-ISI_{k}/\tau )}\right)+{\tilde{F}}_{k-1}\cdot e^{-ISI_{k}/\tau }, \end{array}$$ (7)

where *τ* = 1s acts as the time scale over which the instantaneous firing rate is estimated, weighing each new AP and the previous firing history [21, 31]. The running value of ${\tilde{F}}_{k}$ was compared to a target frequency *F*_*target*_ and employed to define an error signal $e_{k}=F_{target}-{\tilde{F}}_{k}$. This was fed into a Proportional-Integral-Derivative controller (PID), realized via software in LCG, and employed to automatically update in closed-loop the value of the constant holding current

$$\begin{array}{c} I_{k\;holding}=g_{P}\cdot e_{k}+g_{I}\cdot \sum_{i=0}^{k}e_{i}+g_{D}\cdot \left(e_{k}-e_{k-1}\right), \end{array}$$ (8)

where *g*_*P*_, *g*_*I*_, *g*_*D*_ are the proportional, integral, and derivative gains, respectively (i.e., *g*_*P*_ = 0.001 pA/Hz, *g*_*I*_ = 0.1 pA/Hz, *g*_*D*_ = 0 pA/Hz). The value of *F*_*target*_ was also used as the initial value for the estimator ${\tilde{F}}_{0}$, in order to reduce undesired transients. While the output of the PID controller was updated every time an AP was detected, it was held *constant* during the time interval starting from the AP preceding the perturbation pulse to the second spike following it, in contrast to what was done in [30]. In other words, the PID controller was temporarily disconnected, while holding its most recent output, before delivering the external current perturbation required for estimating the PRC, in order to minimize any artifact. A detailed description of the experimental setup is given elsewhere [21].

Our *frequency-clamp* allowed us to rapidly and precisely explore several firing-rates, simply varying *F_target_*. In addition, as in closed-loop the timing of the external current pulse required for the PRC estimation could be precisely chosen in reaction to an AP and after a certain time delay, we optimally synthesized the values of these delays in order to sample the range [0; 1] of *φ* with maximal efficiency (i.e., more uniformly than pseudo-random number generation). We employed a Sobol sequence, first used in [32] and described in [33]: briefly, after online detection of an AP, the external pulse was delivered in a reactive-clamp fashion [34] after a delay *T_i_*, generated as the i-th element of a Sobol sequence (Grey code variant [35], after discarding the initial points). This was repeated at least 1400 times at a rate of one perturbation every 6 APs, independently of the cell’s firing rate.

In an initial set of experiments we compared the PRCs estimated in the same cell or across cells, for fixed firing rate, with and without the PID controller and found no differences. Furthermore the PRCs estimated are in good agreement with those obtained for PCs using a similar method [14], which provides an additional control to our methods. In order to study the influence of the PID controller in our PRC estimates we used the Khaliq and Raman [36] model (see S1 Fig, Discussion and Modeling methods).

### Peak-to-baseline ratio

In order to obtain a concise description of a PRC and to compare those obtained at different AP firing rates, for the same cell and across cells, we adopted the peak-to-baseline ratio *r* as in [14]:

$$\begin{array}{c} r=\frac{\left|m_{l}-m_{e}\right|}{\left|m_{l}\right|+\left|m_{e}\right|}, \end{array}$$ (9)

where *m*_*l*_ and *m*_*e*_ are the values of the late and of the early local extrema (i.e., largest peaks in absolute values) for each of the two halves of *Z*(*φ*) (i.e., in *φ* ∈ [0; 0.5] and in *φ* ∈ [0.5;1]). Note that when *m*_*l*_ and *m*_*e*_ have opposite signs (e.g., as in type II PRCs, [37]), *r* = 1. This also allowed us to concisely quantify the dependency of the PRC shape on the firing rate *F*, by fitting to *r*(*F*) the parameters of a sigmoidal function

$$\begin{array}{c} r\left(F\right)∼{\left(1+e^{-(F-a)/b}\right)}^{-}1. \end{array}$$ (10)

### Modeling

All simulations were performed using the NEURON simulation environment [38]. The simulation code is available on ModelDB or by request from the corresponding author.

**Single-compartment model**. In a first set of simulations, we used the single-compartment, conductance-based PC model described in [36] and available on ModelDB at the URL https://senselab.med.yale.edu/modeldb, accession number 48332. To test the hypothesis that voltage fluctuations endogenously generated by the random opening and closing of ion channels might influence the shape of the PRC, we incorporated channel noise into the model using the method described in [39]. Briefly, the fluctuations induced by channel noise can be accounted for by extending the dynamics of the ionic conductances present in the model according to the equation

$$\begin{array}{c} g\left(t\right)=\bar{g}\;\left[p_{o}\left(t\right)+\sum_{i=1}^{N-1}\eta_{i}\left(t\right)\right], \end{array}$$ (11)

where $\bar{g}$ is the maximal conductance, *p*_*o*_(*t*) is the fraction of open channels (for a deterministic model, $g(t)=\bar{g}\;p_{o}(t))$, *N* is the number of states of the equivalent kinetic scheme and each *η*_*i*_(*t*) is the solution to a stochastic differential equation of the form

$$\begin{array}{c} \tau_{i}{\dot{\eta }}_{i}\left(t\right)=-\eta_{i}\left(t\right)+\sigma_{i}\sqrt{2\tau_{i}}\;\xi_{i}\left(t\right), \end{array}$$ (12)

where *ξ*_*i*_(*t*) is a delta-correlated Gaussian process with zero mean and unitary variance. For the general case of arbitrary kinetic schemes, the *N* − 1 time constants *τ*_*i*_ and standard deviations *σ*_*i*_ are obtained numerically from the *N* × *N* transition matrix of the system that contains the transition rates between all possible states in the kinetic scheme. In the case of the Khaliq-Raman model, this approach was employed only for the resurgent sodium current, which is described by a kinetic scheme that cannot be mapped into the composition of multiple two-state subunits. For all other ionic conductances, the coefficients *τ*_*i*_ and *σ*_*i*_ were analytically calculated using the procedure detailed in [39].

The dimensions of the single compartment were adjusted in order to produce the desired coefficient of variation of the unperturbed spiking pattern. We chose two values of CV, low (around 5%) and high (around 10%), corresponding to values of both length and diameter of 160 and 80 μm, respectively. In the deterministic model, the length and diameter were set to 80 μm and an additional noisy current, modeled as delta-correlated Gaussian white noise, was injected to obtain comparable values of CV.

For the computation of the PRC, we evolved the model until it reached a steady state and then applied pulses of current (0.5 ms duration and 0.5 nA amplitude) at random times with a mean period between perturbations of 2.5 Hz. A constant current offset was injected to vary the baseline firing frequency of the model. In some simulations, this offset current was computed by a PID controller, to replicate and validate in silico the closed-loop technique employed in the experiments.

**Multi-compartment model**. To elucidate whether the presence of an extensive dendritic tree might influence the firing rate dependency of the PRC, we used the De Schutter-Bower model [40, 41].

PRCs were computed without and with ongoing synaptic activity. In both cases, the algorithm employed to compute the PRC differed from that used in the single compartment model and resembled the one adopted in [42, 43]: briefly, after evolving the model until it reached a steady state, we identified two spike times *t*_0_ and *t*_1_ such that the ISI *t*_1_ − *t*_0_ was of the appropriate duration. Then, we simulated the model again until *t*_0_ − 5 ms and saved the full state of the model at this point in time. Finally, starting from *t*_0_ − 5 ms, we evolved the model until *t*_1_ + 10 ms for *N* = 50 trials: current perturbations (0.5 ms duration and 0.2 nA amplitude) were applied at times given by

$$\begin{array}{c} t_{p}^{i}=t_{0}+i\cdot \frac{t_{1}-t_{0}}{N}\;\text{for}\;i=1\;\ldots \;N, \end{array}$$ (13)

where $t_{p}^{i}$ is the perturbation time in the *i*-th trial.

In the case of the model without synaptic inputs we used the PM10 model [40], set the temperature of the simulation to 28°C and injected a somatic current of varying amplitude to span a wide range of firing rates. In the case of the model with synaptic inputs, we used the PM9 model with synapses distributed on the dendritic tree as described in [41], set the temperature to 37°C and fixed the presynaptic excitatory and inhibitory firing rates to 35 and 2 Hz, respectively.

## Results

We characterized the *in vitro* response properties of Purkinje cells (PCs) by patch-clamp electrophysiology, recording from a total of 58 tonically firing PCs in rat cerebellar acute slices. The passive membrane properties of the cells were measured in terms of the input resistance (21.8 ± 4.7 MΩ) and membrane time constant (40.7 ± 19.8 ms). We employed the current-clamp configuration and focused on the input-output relationship between the phase shift Δ*φ*, induced in the cell firing cycle by an external current pulse, and the phase *φ* at which the pulse was repeatedly delivered (Fig. 1; see Materials and Methods). Known as the phase response curve (PRC), this characterization has been increasingly employed to study and classify cellular excitability [11], accompanying conventional descriptions such as, e.g., the frequency-current curve. Previous studies suggested that at least 500 repeated stimulations (i.e., trials) are required to accurately estimate PRCs in tonically firing neurons using direct estimation methods [24]. Here we used 946 to 17278 trials (mean = 2650) to compute 112 PRCs from 42 PCs using the *direct* method.

While firing spontaneously and regularly *in vitro* over long periods, and responding to DC depolarizing or hyperpolarizing holding currents by increasing or decreasing their firing rate, PCs experience substantial firing rate adaptation over very long time scales. Methods to estimate the PRC under transient conditions have been recently developed: [44] however, in order to facilitate the comparison of our results to those of [14] and because the firing rate range that we want to probe is large, we choose to acquire PRCs in a regime of stationary firing. This often leads to discarding a series of stimulation trials in order to avoid an artificially skewed distribution of inter-spike intervals. Similarly, it is often necessary to wait until PCs reach a steady state firing rate before initiating the repeated stimulation protocol. This makes computing several PRCs in the same cell impractical, for instance when one is interested in investigating the effects attributable to the cell’s firing regime, pharmacological manipulation, or recording conditions. We therefore designed and implemented via software a real-time closed-loop control system (Fig. 1A), employed to speed-up convergence to the firing rate steady state and reduce very slow fluctuations. In addition, following [45] we employed a quasi-random Sobol number generator in a reactive-clamp configuration, to sample uniformly and more efficiently the input phase *φ* interval [0; 1]. Using the Khaliq-Raman model (see Methods), we investigated how the closed loop system affects the PRC estimate. Phase response curves estimated with open- or closed-loop methods were strikingly similar (S1 Fig).

This system allowed us to both maintain the cell at the desired target frequency and significantly reduce the time required for a reliable estimation of the PRC. We note that the approach of [44] could equally be used to study the firing rate dependency of the PRCs.

### The PRC dependency on firing rate is smooth

**Fig 2:**
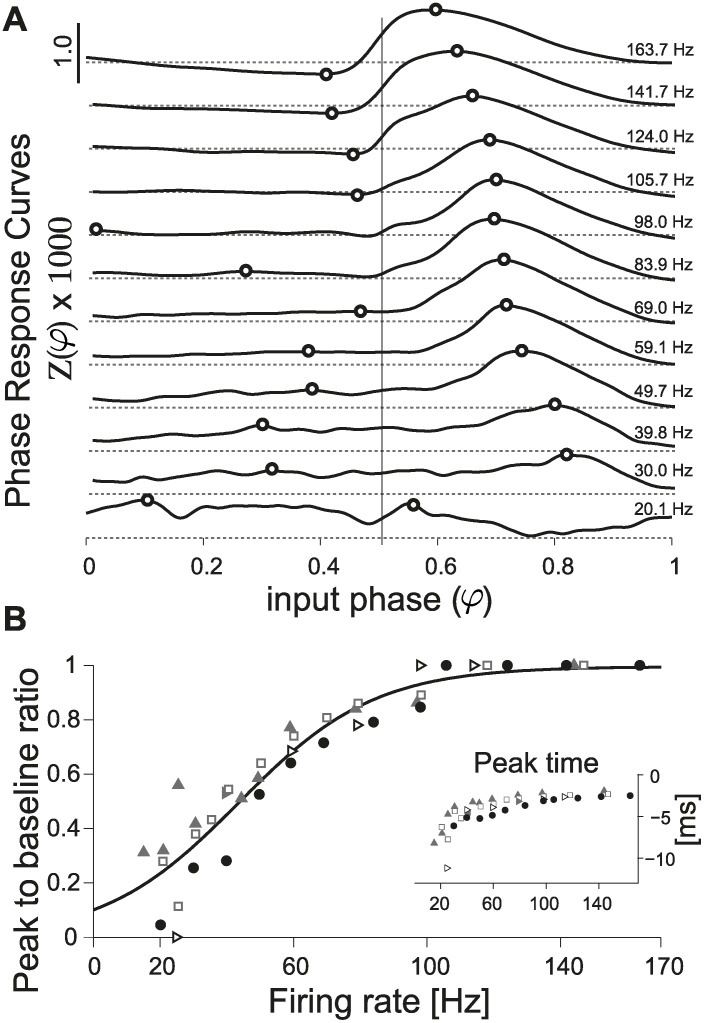
Direct method for the estimation of the PRC. (A) A direct estimate of the PRC, obtained for the same PC, is plotted after data smoothing (see Methods) while altering the cell’s firing rate in the range 20 − 160 Hz): a strong dependency on the firing rate is apparent. The transition from an approximately flat to a phase-dependent PRC profile does not occur abruptly, but smoothly: in each subplot, the horizontal gray dashed lines represent *Z*(*φ*) = 0, while the continuous black thick traces are the PRCs, estimated at distinct firing rates (Fig. 1). Black circles indicate the location of the extrema for each of the two halves of the curves (i.e., in *φ* ∈ [0; 0.5] or [0.5; 1], emphasized by the vertical thin black line), used to concisely characterize the PRC shape according to its peak-to-baseline ratio (see Methods). The graded PRC shape dependency on the firing rate is confirmed in three other PCs (B, markers) and quantified by their peak-to-baseline ratio. The black curve represents the function (1 + *e* ^−(*F*−*a*)/*b*^)^−1^, with best-fit parameters *a* = 44.1, *b* = 20.5. The inset further displays the location of the PRC peak, relative to the time of the AP following the stimulus (i.e., *τ*_*peak*_ = (*τ*_*peak*_ − 1) ⋅ ⟨*ISI*⟩), for the same five cells.

Using our closed-loop system and considering a wide range of firing rates (i.e., 20 − 150 Hz), we could for the first time systematically and extensively investigate how the PRC depends on the firing rate in the very same PCs. Fig. 2A displays 12 PRCs, estimated under stable recording conditions, while repeatedly altering the PC firing rate in a shuffled order. While at low firing rates the profile of the PRC appears relatively flat and independent off the input phase *φ*, at higher rates the profile changes: a late-phase peak (i.e., in the range [0.5; 1]) becomes sharper and shifts to the left, while the average amplitudes in [0; 0.5] decrease. This was quantified for this PC, and four other individual cells, by defining the peak-to-baseline ratio (Fig. 2B; see Methods). This is a measure of the absolute difference between absolute peak values in the ranges [0; 0.5] and [0.5; 1], and it is maximally 1 when these two peaks have different signs. For all individual neurons, the peak-to-baseline ratio increased smoothly in the range of physiological firing rates under consideration, and it could be best fit by a sigmoidal function.

**Fig 3:**
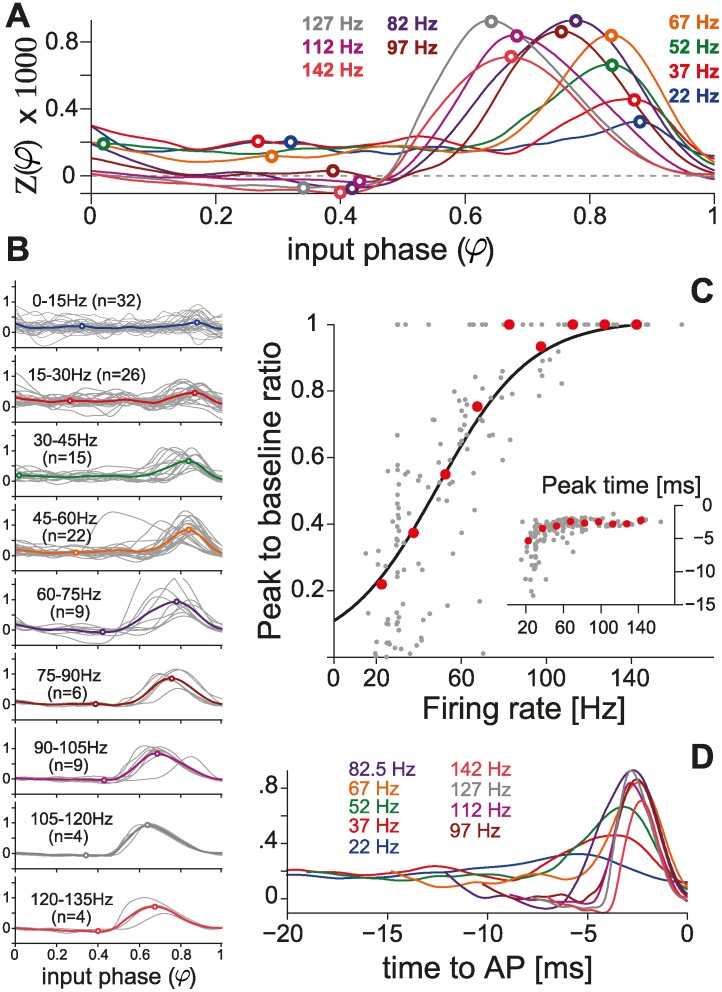
Population summary using the direct method. (A) Population summary obtained over distinct firing rates, by averaging PRCs across individual cells. (B) Individual responses are plotted in gray and pooled according to the corresponding firing rate. Cell numbers are further indicated in parentheses. This summary, quantified by the peak-to-baseline ratio as in Fig. 2C (individual cells, n = 42: gray markers; averages from A: red markers), confirms (C) our observations in single PCs (Fig. 2). The black curve represents the function (1 + *e* ^−(*F*−*a*)/*b*^)^−1^, with best-fit parameters *a* = 47.6, *b* = 21.7 optimized over the set of 42 PCs. The inset further displays the location of the summary PRCs peak, relative to the time of the AP following the stimulus (i.e., *τ*_*peak*_ = (*φ*_*peak*_ − 1) ⋅ ⟨*ISI*⟩), as in Fig. 2B. (D) The average PRCs are equivalently represented as a function of time (i.e., *τ* = (*φ* − 1) ⋅ ⟨*ISI*⟩) for the 20 ms preceding the perturbed AP.

According to the definition of phase *φ*, which is normalized by the average inter-spike interval, the existence of a preferred, rate-independent time-to-spike would correspond to a linear rate-dependence in the phase domain. Since we observed a rate-dependent shift in the PRC late-peak (Figs. 2A and 3A) we asked whether this reflects a time-to-spike preference. To test this possibility we applied the following change of variables *φ* = 1 + *t*_*AP*_/⟨*ISI*⟩, where the *t*_*AP*_ is the relative time to the AP following the stimulus, and plotted the PRC as a function of time, for distinct firing rates (Fig. 3D). The location of the maxima of these plots (Figs. insets in 2B and 3B), displayed a marked dependence on the firing rate in the range [0; 100] Hz while, at very high firing rates, i.e. above 100 Hz, it became rate-independent and equal to 2 ms. This suggests that the rate-dependence of the PRC late-peak (Figs. 2A and 3A) does not result from a rate-independent time-to-spike preference. Our observations are not changed when using the truncated Gaussian method [45] as illustrated in S2 Fig.

**Fig 4:**
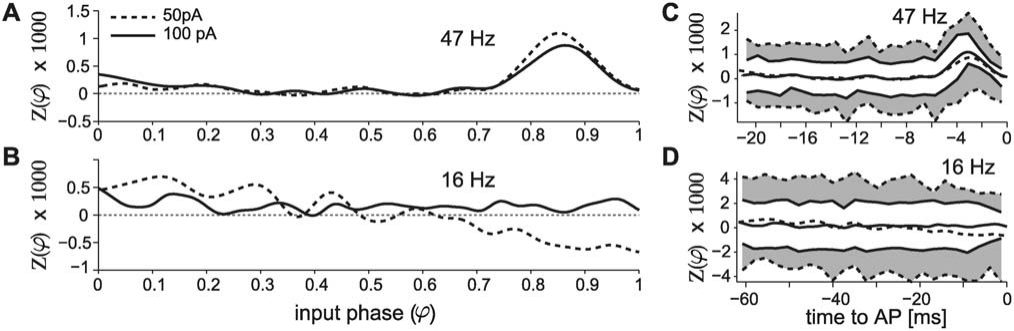
Signal to noise ratio in PRC estimates. PRCs were smoothed and normalized by the total charge of the injected pulse *Q* = *I*_*pulse*_ ⋅ *T*_*pulse*_. Increasing the amplitude *I*_*pulse*_ increased the estimates confidence (A-B), reducing the standard deviation of the PRC raw data points, especially as PCs fire at low firing rates. The plots display the PRC estimates obtained without the PID controller for *I*_*pulse*_ = 50 and 100 pA, in the same PC over a comparable number of stimulation trials (i.e., 1911 and 1338 at high firing rates, and 3361 and 3350 at low firing rates, respectively). The smoothed PRCs and their 68% confidence intervals (C-D) are plotted, as a function of time (i.e., *τ* = (*φ* − 1) ⋅ ⟨*ISI*⟩) by the lines and shaded areas (i.e., dashed/gray for 50 pA and continuous/white for 100 pA).

In an initial set of experiments, we wondered whether the rather flat profile of the PRC observed at low firing rates was an intrinsic property of the cells. Very weak input stimuli may be in fact effectively ignored by the cell and result in a phase-independent PRC profile. The benefits of systematically acquiring PRCs, across distinct conditions along stable recording sessions, were again exploited: we injected in the same PCs an external input with different amplitudes, over distinct firing rates. An example of such experiments is reported in panels A-B of Fig. 4, where the impact of the pulse amplitude is apparent: the signal-to-noise ratio increases for stronger pulses, while the PRC is indeed phase-independent. Fig. 4C-D further visualize graphically the 68% confidence intervals of the PRC estimates, revealing an almost two fold reduction when using doubled pulse amplitudes.

### Population summary

Across the entire data set collected, several amplitudes of the external stimuli and various firing rates were explored over 42 PCs. When expressed in terms of a population summary, the PRCs confirmed our previous observation (Fig. 3A). When pooling the PRCs obtained across cells, in 15 Hz-wide bins, according to the PC firing rate at which each curve was measured, a moderate amount of variability was observed (Fig. 3B). The overall quantification in terms of the peak-to-baseline ratio, already discussed in Fig. 2B, is comparable to that measured for single cells, revealing and confirming the same marked smooth dependency on the firing rate (Fig. 3C). As in Fig. 2B, the inset of Fig. 3C displays the location in time of the peak of the PRC against the firing rate, and the average PRCs were also plotted as a function of time to the next AP (Fig. 3D). While slightly noisier than the data acquired within the same PCs, this evidence prompts us to exclude that the late-peak in the PRC is an artifact of the phase normalization.

### Indirect methods for PRC estimation

**Fig 5:**
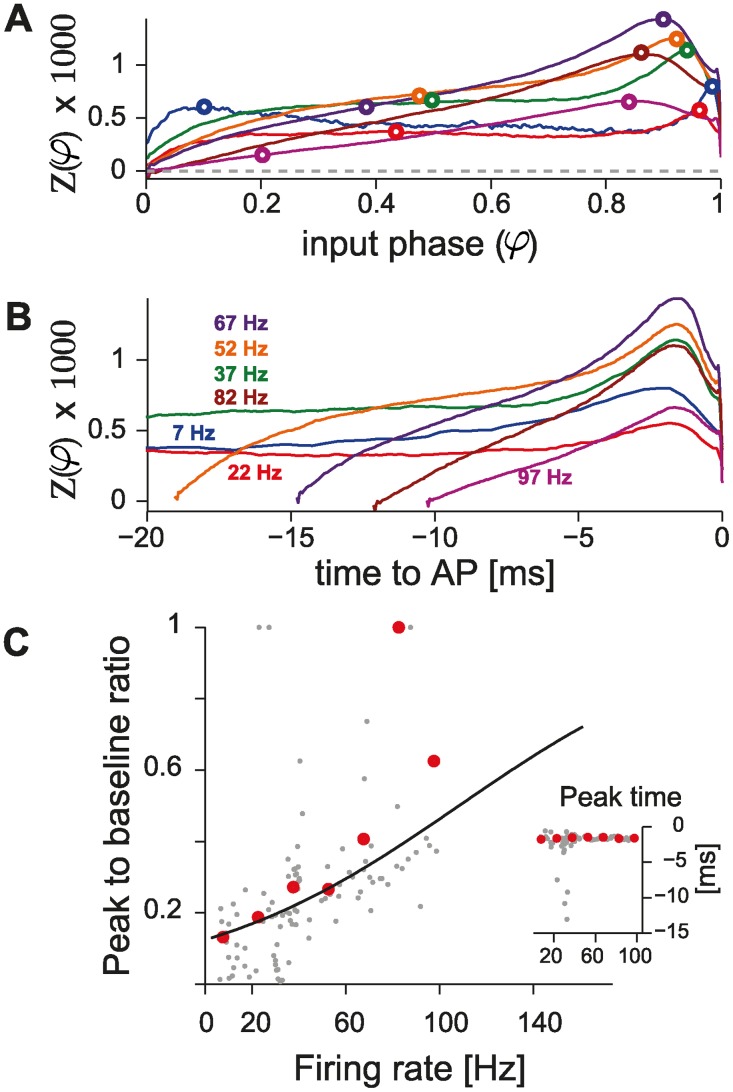
Population summary using the WSTA (indirect) method. (A) Population summary obtained over distinct firing rates, obtained averaging across 95 PRCs, binned for similar firing rates and obtained from 16 PCs. As in Fig. 3D, the average PRCs are equivalently represented (B) as a function of time (i.e., *τ* = (*φ* − 1) ⋅ ⟨*ISI*⟩) for the 20 ms preceding the perturbed AP. (C) The peak-to-baseline ratio once more confirms the observations obtained in Figs. 2, 4 and 3 (individual cells, *n* = 16: gray markers; averages from A: red markers). The black curve represents the function (1 + *e* ^−(*F*−*a*)/*b*^)^−1^, with best-fit parameters *a* = 105.6, *b* = 55.8 optimized over the set of individual PCs. The inset displays the location of the PRC peak, relative to the time of the AP following the stimulus (i.e., *τ*_*peak*_ = (*φ*_*peak*_ − 1) ⋅ ⟨*ISI*⟩), for the same cells, as in Figs. 2B and 3C.

We further employed an alternative method for PRC estimation, based on the weighted spike triggered average (WSTA) (see Materials and Methods and [13] for a review). We computed 95 PRCs in 16 PCs, with firing rates below 100 Hz. The average PRC profiles, obtained from pooled data as in Fig. 3B across several firing rates, are shown in Fig. 5A-B were the PRCs are plotted both as a function of the phase of the time to the next AP. As for Figs. 2 and 3, performing a quantification based on the peak-to-baseline ratio revealed a qualitatively similar frequency dependence of the PRC profile on the PC firing rate.

Quantitatively, however, the dependency on the firing rate observed with WSTA methods did not match that obtained by the direct estimation method of the PRCs. To some extent, we attribute this inaccuracy to the WSTA method: while for direct methods the stationarity of the firing rate could be precisely monitored and controlled in closed-loop, indirect methods do not allow the same precision, as they require the injection of a noisy current waveform that elicits a train of APs with some variability. It should be noted that a method has been developed recently that allows a more accurate estimation of PRCs while using fewer spikes [44, 46]. We did not employ this method since we were mostly concerned with validating the results obtained with the direct method. Nonetheless, the presence of a late peak in the PRCs at high firing rates confirms that our observations do not depend on the PRC estimation method in use. When referred to the time of the next AP, the location of the PRC peak averaged to a value of −1.7 ± 0.3 ms (Fig. 5C, inset), with the exception of some (n = 4) cases where the PC fired at low rates and the PRC did not exhibit a peak at late phases.

### Computational modeling

**Fig 6:**
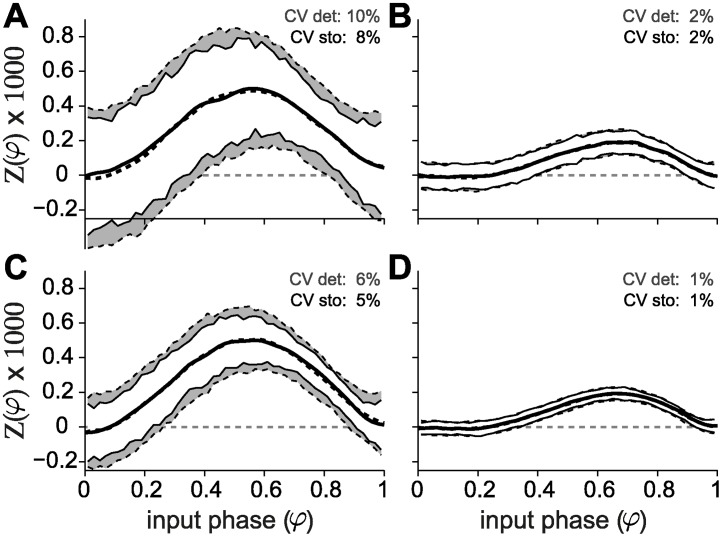
Stochastic channel noise is not responsible for the firing rate dependency of the PRC. The PRCs of the model incorporating channel noise (gray curves) are strikingly similar to those of the deterministic model (black curves). The variability of the spike trains does not affect the curves either at low (A and C) or high (B and D) firing rates (left and right columns, respectively). The membrane area of the model with channel noise was chosen to obtain the desired value of CV at low firing rate (around 10% for A, and 5% for C) and that same area was used for the simulations at high firing rate (panels B and D). Comparable values of CV were obtained in the deterministic model by changing the magnitude of the fluctuations of the injected current. For a given perturbation size, the model is less sensitive at high firing rates (B and D).

Active conductances are thought to modulate the shape of the PRC, and therefore computational modeling [7, 47, 48] could be a powerful tool to dissect the ionic bases of the PRC. The conductance-based models developed in [36, 49] to recapitulate several experimental observations in Purkinje cells were used in [14] to attempt to reproduce the PRC shape and in particular its dependency on the firing rate. Such attempts have proved largely unsuccessful and up to today a model able to reproduce the rate dependency observed experimentally remains elusive. Having observed a rate dependency in the CV of PCs in our experimental data (S1 Fig, panel C) we asked whether spiking variability could account for the flat PRC profile observed at low firing rates. To test this hypothesis, we employed the Khaliq-Raman model [36] and computed PRCs at low and high firing rates. We compared the results obtained when spiking variability was introduced via additive noise fluctuations (i.e., by injecting a noisy current into the model neuron) with those obtained in the presence of endogenously generated channel noise (see Methods). The results are shown in Fig. 6: solid lines denote the stochastic model, whereas dashed lines represent the deterministic model with external noisy current. In both conditions, the model is more sensitive to perturbations when firing at low rates (left panels (A and C)), which translates both to a larger amplitude of the PRC and to a higher CV, for the same number of channels (and therefore magnitude of the internally generated fluctuations) or variance of the noisy current. This result holds true both in the case of low and high variability, as exemplified by the top and bottom panels, respectively. Importantly, in all four conditions tested (low and high firing rate, low and high CV), the PRCs of the stochastic and deterministic models are strikingly similar and, for a given firing rate, do not depend on the amount of endogenous or exogenous noise. Furthermore, the models fail at reproducing the flat PRCs observed in vitro at low firing rates and the greater sensitivity to perturbation amplitude at high firing rates typical of the experiments.

In order to elucidate whether the presence of the large dendritic tree characteristic of Purkinje cells could be responsible for the flat PRCs observed in vitro at low firing rates, we employed a detailed, multi-compartmental model of a Purkinje cell [40]. We compute PRCs spanning firing rates in the range 33 to 77 Hz (Fig. S3 Fig) and observed a decrease in *Z*(*φ*) for perturbations delivered in the early phase of the spiking cycle. Additionally, in our simulations early-phase *Z*(*φ*) became negative for firing rates above approximately 50 Hz. The shape at late phases remained unchanged even for high firing rates, which is in contrast with the experiments (compare S3 Fig with Fig. 2), in which the late peak drifts towards the middle of the PRC. The peak-to-baseline ratio of the multi-compartmental model seems to capture some of the experimentally observed features of the PRC, although interpretation of these results requires great care, see the Discussion. Purkinje cells are endowed with very large dendritic trees with thousands of contacts from parallel fibers, which might provide a dendritic load—even in the relatively quiet conditions of the slice preparation—by means of spontaneous synaptic release. To test whether distributed synaptic inputs might influence the shape of the PRC, we delivered random synaptic inputs to the dendrites of the model neuron. We found that, even when the instantaneous rates of inhibitory and excitatory inputs were relatively stable, synaptic inputs greatly affected the shape of the PRC (S3 Fig, panel A). These simulations suggest that a large portion of the variability observed in the PRC shape in Purkinje cells, even for the same firing rate (Fig. 3B), could be attributable to spontaneous activation of dendritic conductances, given that even similar presynaptic patterns can result in very different PRCs (S3 Fig, panels B and C). We conclude that realistic dendritic activation interferes with the PRC measurement, and, even though for some selected trials (see Methods) the model seems able to partly reproduce the experimental observations, the PRCs computed in these conditions are extremely variable.

## Discussion

Phoka and colleagues recently discovered and described the heterogeneity of PRCs in murine PCs in vitro, depending on their spontaneous firing rate. However, while their main focus was to introduce and test a novel technique for the PRC estimation, their experimental data set consisted of a small population of 16 cells. In addition, only for a very small number of PCs, could the response of the same cell be investigated under stable recording conditions, while studying two firing regimes (i.e., low and high firing rates). In fact, despite the increased accuracy, the PRC estimation technique they introduced requires a high number of stimulation trials for each recorded cell: for instance, at least 10000 trials were used in Fig. 5 of [14], which correspond to ∼ 1 h when sampling low firing rates. Aiming at replicating their intriguing discovery, we immediately realized that this made rather impractical a more systematic exploration of a wider set of firing rates in the same cell: stable recording conditions could not be maintained except under extraordinary circumstances. Our efforts confirm and extend the previous report, aiming at a consistent, accurate, and conclusive evaluation of the PRC in PCs. We specifically implemented a real-time system composed of a closed-loop controller of the cell instantaneous firing rate. This allowed us to compensate for the intrinsic variability in the firing rate, the extremely long transients before steady-state, and the very slow drifts. Ultimately, we could obtain accurate PRC estimates with fewer trials and over reduced intervals of time and thus explore a broad range of firing frequencies (i.e., 20 − 150 Hz). Of course, the controller was disabled each time a stimulation was applied, to decrease the chance of introducing artifacts. Our data clearly and unambiguously demonstrate a strong modulation in the shape of the PRC by the firing rate, in all PCs we recorded and irrespective of the estimation method and the amplitude of the external perturbation pulse (Figs. 2–5). In particular, PCs smoothly alter their dynamical response properties, from non-ideal integrators at low firing rate (i.e., ∼ 20 Hz) to phase-dependent integrators at higher firing rates (i.e., ∼ 80 Hz) (Figs. 2, 3 and 5 and also S5 Fig).

### PRC estimation methods

In [50] and [8], less than 180 trials were used to compute the PRC of individual cortical neurons by direct methods, although current pulses were stronger in amplitude and longer in duration than those used in PCs in [14]. In addition, cortical neurons fire APs only upon external holding current injection. In comparison, PRCs of PCs are generally very noisy and require a substantially higher number of trials, however employing larger pulse amplitude reduces PRC raw data variability and increases confidence of its estimate (Fig. 4). Along the lines of improving the confidence of the PRC estimates, novel (e.g., indirect) methods and closed-loop experimental protocol recently received increasing attention [13, 29–32, 51]. Our work has been greatly inspired by those previous proposals as we combined some of the existing paradigms together, benefiting from advantages of the (corrected) direct method. This was an important design element in our experiments, enabling to study PCs across a variety of firing rates and ultimately helped to unambiguously conclude on a smooth transition of the PRC shape, and improved the firing rate resolution. While PRCs estimated by the indirect method did not perfectly match those obtained by the (corrected) direct method, smooth firing rate dependency of PRCs was nonetheless confirmed.

We asked whether the PID controller might affect the estimation of the PRC. To clarify this point, we compared open- with closed-loop estimates in a single-compartment conductance-based model of a Purkinje cell [36] (S1 Fig, panel A). The usage of a PID controller resulted in a slight increase in the coefficient of variation of the unperturbed trials (approximately 0.07% increase) while leaving the curves almost unchanged. We observed no increase in the variability of inter-spike intervals in the experiments in which the PID controller was employed (filled markers in S1 Fig, panel B).

### Conductance-based neuron modeling

Computer simulation of biophysically accurate models of neuronal excitability is, in the context of the study of intrinsic response properties, a powerful complementary tool to cellular electrophysiology. For PCs, mathematical modeling might help identifying candidate ionic bases for the observed firing rate dependency of PRCs. In [14], several attempts to reproduce the firing rate dependency of the PRC were described, in particular employing the conductance-based models proposed by Khaliq and Raman [36] and by Akemann and Knöpfel [49]: none reproduced the phase-independent PRC profile at low firing rates and the overall firing rate modulation. In our experimental data set, the inter-spike intervals distributions displayed a significant inverse correlation of the coefficient of variation (CV) with the firing rate (i.e., Pearson’s *r* = −0.4, *p* < 10^−6^, and a slope of −0.25/kHz—S1 Fig, panel B) regardless of whether open- or closed-loop methods used.

We first simulated the deterministic KR model, in which we injected a noisy current given by

$$\begin{array}{c} I\left(t\right)=I_{m}+I_{s}\xi \left(t\right), \end{array}$$ (14)

where *I*_*m*_ is the mean of the injected current and was used to set the mean firing rate of the model cell, *I*_*s*_ is the standard deviation and *ξ*(*t*) is delta-correlated Gaussian white noise. The value of *I*_*s*_ was chosen in order to obtain the desired variability of the ISIs (corresponding to a CV between 5 and 10%). The results are shown in Fig. 6 (black traces), for high and low firing rates (left and right columns, respectively). Note how the amount of variability in the spiking response primarily influences the dispersion of the points in the PRC but not its shape at either high or low firing rates.

We then speculated that stochastic fluctuations of voltage-gated channels could play a role in disrupting phase-preference at low firing rates, given the (inverse) rate-dependent AP jitter variance. An augmented version of the Khaliq-Raman PC model, with stochastic channel gating [39] and geometry adjusted to reproduce realistic inter-spike interval distributions was used to investigate whether stochastic fluctuations of ionic channels could be responsible for the observed phenomena. This was of particular interest since the Khaliq-Raman model describes also potassium channels with large single-channel conductance (i.e., > 200 nS) [36] where channel noise could play a larger role. We observed no difference between the stochastic and the deterministic models, even though the CV was reduced at high firing rates in the stochastic model (for the same cell size and channel density). Nonetheless, biophysically accurate (i.e., voltage-dependent) channel flickering had a similar effect on the PRC as what was obtained by additive Gaussian noise current injection, in [14] and our simulations.

We note that, in response to external current injection, the Khaliq-Raman model fires at arbitrarily low firing rates, implying a type I excitability. Given the known relationship between excitability type and PRC profile [5], it is surprising that the model displays a fully positive PRC with a peak around *φ* = 0.5 at low firing rates, which then turns into a slightly more bimodal profile as the firing rate is increased, in a way that is reminiscent of a type II PRC, with a peak around *φ* = 0.7 and a slightly negative component at early phases—see Fig. 6 and S3 Fig of [14] (the model is available from ModelDB [23] at http://senselab.med.yale.edu/modeldb (accession number 155735).

The rate dependent transition in this model is not quantifiable by the peak-to-baseline since there are no distinct peaks at low rates that can be distinguished for early and late phases. This has to our knowledge not been previously highlighted and suggests that the Khaliq-Raman (as well as the Akemann and Knöpfel) model may provide grounds for further investigations on the modulation of the PRC by the firing rate. Another overlooked point in previous studies is that as the firing rate is increased the sensitivity of the neuron to the perturbation decreases dramatically (Fig. 6—low versus high firing rate). This is in disagreement with what was observed in the experiments and illustrates how a biophysically realistic model can fail to describe simple neuronal behaviors while taking in consideration complex channel kinetics.

We then asked whether the presence of a dendritic tree might be responsible for the observed phenomena, and used a morphologically detailed multi-compartmental model [40] to test our hypothesis. Having shown that the shape of the PRC is not affected by the CV of the ISI, we first simulated the model with the injection of a DC current (i.e., *I*_*s*_ = 0 in Eq. 14, leading to a CV = 0) of varying amplitude to span several firing rates. Additionally, we set the simulation temperature to 28°C, in order to span a broader range of steady state firing frequencies, particularly in the low end of the spectrum. While the model failed to reproduce the shape of experimental PRCs at low firing rates, it produced curves characterized by a peak at early phases that becomes negative as the firing rate is increased (S3 Fig, panel A). This behavior results in lower peak to baseline values at low firing rates (S3 Fig, panel B) even though the shape of the curves does not resemble that of the experimental PRCs. Finally, similarly to the KR model, but in contrast with the experiments, the model is more sensitive to perturbations at low firing rates.

One caveat of this study (S3 Fig, panel A) is that we did not compare the PRCs obtained with those of a single compartment model with the same channel descriptions. However this study’s focus is on whether the flat PRC at low rates observed *in vitro* could be explained by the presence of the dendritic tree, and not in the effects of complex dendritic morphologies on somatic PRCs. It is then valid to conclude that the PRCs firing rate dependency in PCs can not be explained by a complex model with realistic morphology.

It is plausible that a detailed model without dendritic load is not a good approximation of the experimental condition due to e.g. spontaneous synaptic release (even though GABA_A_ inhibition to PCs was blocked). Furthermore, we wanted to conduct simulations with the correct temperature factor since temperature severely affects channel dynamics. In order to simultaneously add dendritic load and increase the temperature of the model to 37°C, we introduced synaptic conductances as described in [41], see Methods. Under these conditions, the model fires irregularly so we focused in computing PRCs for selected inter-spike intervals, for which the expected ISI was known (see Methods). Despite our focus on inter-spike intervals that did not have abrupt conductance fluctuations (S3 Fig, panel B), even a slight fluctuation in the rate of dendritic events severely affected the shape of the somatic PRCs (S3 Fig, panel C). Nevertheless for selected inter-spike intervals, the model with dendritic load could approximate the strong difference in PRC shape at low versus higher rates (S3 Fig, panel A). However, given the high sensitivity to dendritic conductances, care should be taken when interpreting this curves as discussed above. Finally, for questions related to network dynamics, network simulations using the PRCs measured in this study could result in more accurate predictions than simulations with conductance based models that do not accurately reproduce the rate dependency of the PRC in the PC.

### Implications for information processing

Considering a framework of elementary computation, we discuss the most immediate consequence of a firing rate-dependent PRC.

In the particular case the Purkinje Cell, the firing rate dependency of the PRC suggests that the cell can act as a perfect integrator at low firing rates and as a phase dependent integrator at high firing rates. The cell would then favour a rate code at low firing rates and a time based code at high firing rates which is a profound implication for information processing in the cerebellum. It has been suggested that neuronal coding depends both on the operating mode and the properties of the stimulus [52], therefore the fact that intracellular properties favor different coding schemes is relevant only if the inputs to the neurons are adjuvant to this hypothesis. It turns out that, a recent study that looked at the relation between population coding by PCs and saccadic eye movements found that the same PC can exhibit multiplexed spike coding, suggesting that despite the reduced level of overall correlation between PC firing rates a small fraction of the spikes can couple to the network activity and report different behavioral information [53]. Our study suggests that the intrinsic properties of PCs favor multiplexed information coding and that there is a smooth dependency on the firing rate of how sensitive PCs are to synchronous inputs.

The phase response curve allows us to discuss the impact of single cell properties on network-level phenomena, as a putative way to relay strong inhibition downstream (by synchronisation of PCs). Furthermore, in the cerebellar microcircuitry, it was shown that the interplay between the activation of parallel fibers and of interneurons affects PCs firing, and was associated with a variety of motor and sensory tasks [54, 55]. Moreover, the incoming information is likely to be processed and conveyed downstream by means of the synchronization of several PCs [18] and high frequency synchrony has been reported in the Purkinje cell layer [17] and has been associated with PC collaterals. We provide a thorough characterization of single cell properties that might be involved in synchronization, hence contributing for the observed phenomena. It is in this perspective that the study of PRC of PCs is of great relevance, as it provides insights in the synchronization mechanisms of neuronal populations. The fact that Purkinje cell’s phase response curve smoothly transitions from phase independent at low firing rates to highly bimodal at high firing rates could then be used as an intrinsic mechanism to modulate the contribution of individual inputs to spiking synchrony across Purkinje cells.

## Supporting Information

S1 Fig The PID controller does not interfere with the PRC estimate. (A) The Khaliq-Raman model neuron was simulated with or without a closed-loop (PID) regulation of its firing rate, as in the experiments. The model PRC was estimated as in Fig. 3 and studied in four conditions: low or high firing rate and with or without PID. The PRC estimates using the PID are strikingly similar to those estimated using the open-loop method. (B) The CV of the experiments with PID (filled markers) are comparable to that of the experiments without PID (open markers).
(EPS)

S2 Fig PRCs estimated with the truncated Gaussian method. Same as Fig. 3 for the truncated Gaussian method. (A) Population summary obtained over distinct firing rates, by averaging PRCs across individual cells. (B) Individual responses are plotted in gray and pooled according to the corresponding firing rate. Cell numbers are further indicated in parentheses. This summary, quantified by the peak-to-baseline ratio as in Fig. 2C (individual cells, n = 42: gray markers; averages from A: red markers), confirms (C) our observations in single PCs (Fig. 2). The black curve represents the function (1 + *e* ^−(*F*−*a*)/*b*^)^−1^, with best-fit parameters *a* = 50.5, *b* = 24.2 optimized over the set of 42 PCs. The inset further displays the location of the summary PRCs peak, relative to the time of the AP following the stimulus (i.e., *τ*_*peak*_ = (*φ*_*peak*_ − 1) ⋅ ⟨*ISI*⟩), as in Fig. 2B. (D) The average PRCs are equivalently represented as a function of time (i.e., *τ* = (*φ* − 1) ⋅ ⟨*ISI*⟩) for the 20 ms preceding the perturbed AP.
(EPS)

S3 Fig PRCs and peak-to-baseline ratio as a function of firing rate in a multi-compartmental model of Purkinje cell. (A) Direct estimate of the PRC, obtained using a step of current to control the firing rate of the cell (data analysed as in Fig. 2). Unlike what was seen in the experiments, the amplitude of *Z*(*φ*) is bigger for lower rates suggesting that the same perturbation pulse advances a bigger percentage of the *ISI* at low firing rates. A small peak is observed at early (B) The peak at early phases induces a decrease in the peak-to-baseline ratio (the black curve represents the function (1 + *e* ^−(*F*−*a*)/*b*^)^−1^, with best-fit parameters *a* = 19.0, *b* = 9.5) that however is not indicative of a flat PRC. Differently from the experiments, Z(phi) is larger at low firing rates, implying that the phase advance, in response to the same perturbation, is greater at lower firing rates.
(EPS)

S4 Fig PRCs computed for a highly detailed computational model of a Purkinje cell with synaptic activation in the dendritic tree. (A) Direct estimate of the PRC, obtained using a dendritic activation to control the firing rate of the cell. (B) Instantaneous rate of presynaptic firing rate during two inter-spike intervals of comparable duration (black and gray). Excitatory firing rate are represented by full lines and inhibitory firing rate by dashed lines. (C) Phase responses obtained from delivering perturbations during two (black and gray) inter-spike intervals of comparable duration. Dendritic conductances can greatly affect the shape of the PRC.
(EPS)

S5 Fig The rate-dependent transition of the PRCs of PCs from perfect integrator to phase dependent integrator, occurs without abrupt transitions. The value of *Z*(*φ* = 0.3) as a function of firing rate, both across PCs (open black circles) and for the same cell (filled gray squares). A gradual transition from positive to more negative values is apparent suggesting a smooth transition. The red markers represent the mean of all experiments. Black and gray curves the linear fit for all experiments and for the single cell respectively.
(EPS)

## References

[1] Fernandez FR, Engbers JDT, Turner RW (2007) Firing dynamics of cerebellar purkinje cells. Journal of neurophysiology 98: 278–94. 10.1152/jn.00306.2007 17493923

[2] Llinás R, Sugimori M (1980) Electrophysiological properties of in vitro purkinje cell dendrites in mammalian cerebellar slices. The Journal of Physiology 305: 197–213. 10.1113/jphysiol.1980.sp013358 7441553 PMC1282967

[3] Llinás R, Sugimori M (1980) Electrophysiological properties of in vitro purkinje cell somata in mammalian cerebellar slices. The Journal of Physiology 305: 171–195. 10.1113/jphysiol.1980.sp013357 7441552 PMC1282966

[4] Loewenstein Y, Mahon S, Chadderton P, Kitamura K, Sompolinsky H, et al (2005) Bistability of cerebellar purkinje cells modulated by sensory stimulation. Nature neuroscience 8: 202–211. 10.1038/nn1393 15665875

[5] Ermentrout B (1996) Type i membranes, phase resetting curves, and synchrony. Neural computation 8: 979–1001. 10.1162/neco.1996.8.5.979 8697231

[6] Ermentrout GB, Galán RF, Urban NN (2007) Relating neural dynamics to neural coding. Physical Review Letters 99: 248103 10.1103/PhysRevLett.99.248103 18233494 PMC2533709

[7] Gutkin BS, Ermentrout GB, Reyes AD (2005) Phase-response curves give the responses of neurons to transient inputs. Journal of Neurophysiology 94: 1623–1635. 10.1152/jn.00359.2004 15829595

[8] Mancilla JG, Lewis TJ, Pinto DJ, Rinzel J, Connors BW (2007) Synchronization of electrically coupled pairs of inhibitory interneurons in neocortex. Journal of Neuroscience 27: 2058–2073. 10.1523/JNEUROSCI.2715-06.2007 17314301 PMC6673558

[9] Smeal RM, Ermentrout GB, White JA (2010) Phase-response curves and synchronized neural networks. Philosophical Transactions of the Royal Society B: Biological Sciences 365: 2407–2422. 10.1098/rstb.2009.0292 PMC2894948 20603361

[10] Winfree AT (1967) Biological rhythms and the behavior of populations of coupled oscillators. Journal of Theoretical Biology 16: 15–42. 10.1016/0022-5193(67)90051-3 6035757

[11] Schultheiss NW, Prinz AA, Butera RJ (2011) Phase Response Curves in Neuroscience. Springer Science, 518 pp.

[12] Hong S, Robberechts Q, De Schutter E (2012) Efficient estimation of phase-response curves via compressive sensing. Journal of neurophysiology 108: 2069–81. 10.1152/jn.00919.2011 22723680

[13] Torben-Nielsen B, Uusisaari M, Stiefel KM (2010) A comparison of methods to determine neuronal phase-response curves. Frontiers in Neuroinformatics 4: 6 10.3389/fninf.2010.00006 20431724 PMC2861477

[14] Phoka E, Cuntz H, Roth A, Häusser M (2010) A new approach for determining phase response curves reveals that purkinje cells can act as perfect integrators. PLoS Computational Biology 6: 665–678. 10.1371/journal.pcbi.1000768 PMC2861707 20442875

[15] Tsubo Y, Takada M, Reyes AD, Fukai T (2007) Layer and frequency dependencies of phase response properties of pyramidal neurons in rat motor cortex. European Journal of Neuroscience 25: 3429–3441. 10.1111/j.1460-9568.2007.05579.x 17553012

[16] Hong S, Ratté S, Prescott Sa, De Schutter E (2012) Single neuron firing properties impact correlation-based population coding. The Journal of neuroscience: the official journal of the Society for Neuroscience 32: 1413–28. 10.1523/JNEUROSCI.3735-11.2012 22279226 PMC3571732

[17] de Solages C, Szapiro G, Brunel N, Hakim V, Isope P, et al (2008) High-frequency organization and synchrony of activity in the purkinje cell layer of the cerebellum. Neuron 58: 775–788. 10.1016/j.neuron.2008.05.008 18549788

[18] Person AL, Raman IM (2011) Purkinje neuron synchrony elicits time-locked spiking in the cerebellar nuclei. Nature 481: 502–505. 10.1038/nature10732 22198670 PMC3268051

[19] Robberechts Q, Wijnants M, Giugliano M, De Schutter E (2010) Long-term depression at parallel fiber to golgi cell synapses. Journal of Neurophysiology 104: 3413–23. 10.1152/jn.00030.2010 20861429 PMC3007626

[20] Davie JT, Kole MHP, Letzkus JJ, Rancz EA, Spruston N, et al (2006) Dendritic patch-clamp recording. Nature Protocols 1: 1235–1247. 10.1038/nprot.2006.164 17406407 PMC7616975

[21] Linaro D, Couto J, Giugliano M (2014) Command-line cellular electrophysiology for conventional and real-time closed-loop experiments. The Journal of Neuroscience Methods 230: 5–19. 10.1016/j.jneumeth.2014.04.003 24769169

[22] Brette R, Piwkowska Z, Monier C, Rudolph-Lilith M, Fournier J, et al (2008) High-resolution intracellular recordings using a real-time computational model of the electrode. Neuron 59: 379–391. 10.1016/j.neuron.2008.06.021 18701064

[23] Hines M, Morse T, Migliore M (2004) Modeldb: a database to support computational neuroscience. Journal of Computational Neuroscience 17: 7–11. 10.1023/B:JCNS.0000023869.22017.2e 15218350 PMC3732827

[24] Galán RF, Ermentrout GB, Urban NN (2005) Efficient estimation of phase-resetting curves in real neurons and its significance for neural-network modeling. Physical review letters 94: 158101 10.1103/PhysRevLett.94.158101 15904191 PMC1410718

[25] Achuthan S, Butera RJ, Canavier CC (2011) Synaptic and intrinsic determinants of the phase resetting curve for weak coupling. J Comput Neurosci 30: 373–90. 10.1007/s10827-010-0264-1 20700637 PMC3059351

[26] Cox D, Miller H (1965) The Theory of Stochastic Processes. Methuen and Company.

[27] Ota K, Nomura M, Aoyagi T (2009) Weighted spike-triggered average of a fluctuating stimulus yielding the phase response curve. Phys Rev Lett 103: 024101 10.1103/PhysRevLett.103.024101 19659207

[28] Bowman A, Azzalini A (1997) Applied Smoothing Techniques for Data Analysis: The Kernel Approach with S-Plus Illustrations (Oxford Statistical Science Series). Oxford University Press, USA.

[29] Netoff TI, Banks MI, Dorval AD, Acker CD, Haas JS, et al (2005) Synchronization in hybrid neuronal networks of the hippocampal formation. Journal of neurophysiology 93: 1197–208. 10.1152/jn.00982.2004 15525802

[30] Miranda-Domínguez O, Gonia J, Netoff TI (2010) Firing rate control of a neuron using a linear proportional-integral controller. Journal of Neural Engineering 7: 066004 10.1088/1741-2560/7/6/066004 20975212

[31] Wallach A, Eytan D, Gal A, Zrenner C, Marom S (2011) Neuronal response clamp. Frontiers in Neuroengineering 4: 1–10. 10.3389/fneng.2011.00003 21519391 PMC3078750

[32] Netoff TI, Acker CD, Bettencourt JC, White JA (2005) Beyond two-cell networks: experimental measurement of neuronal responses to multiple synaptic inputs. Journal of Computational Neuroscience 18: 287–295. 10.1007/s10827-005-0336-9 15830165

[33] Netoff T, Schwemmer MA, Lewis TJ (2012) Experimentally estimating phase response curves of neurons: theoretical and practical issues In: Phase Response Curves in Neuroscience, Springer pp. 95–129.

[34] Fellous JM, Sejnowski TJ (2003) Regulation of persistent activity by background inhibition in an in vitro model of a cortical microcircuit. Cerebral Cortex 13: 1232–1241. 10.1093/cercor/bhg098 14576214 PMC2928820

[35] Press WH (2007) Numerical Recipes 3rd Edition: The Art of Scientific Computing. Cambridge University Press.

[36] Khaliq ZM, Gouwens NW, Raman IM (2003) The contribution of resurgent sodium current to high-frequency firing in purkinje neurons: an experimental and modeling study. Journal of Neuroscience 23: 4899–4912. 12832512 10.1523/JNEUROSCI.23-12-04899.2003 PMC6741194

[37] Koch C, Segev I (1989) Methods in Neuronal Modeling: From Synapses to Networks (Computational Neuroscience). The MIT Press.

[38] Hines ML, Carnevale NT (2001) Neuron: a tool for neuroscientists. Neuroscientist 7: 123–35. 10.1177/107385840100700207 11496923

[39] Linaro D, Storace M, Giugliano M (2011) Accurate and fast simulation of channel noise in conductance-based model neurons by diffusion approximation. PLoS Computational Biology 7: e1001102 10.1371/journal.pcbi.1001102 21423712 PMC3053314

[40] De Schutter E, Bower JM (1994) An active membrane model of the cerebellar purkinje cell. i. simulation of current clamps in slice. Journal of Neurophysiology 71: 375–400. 7512629 10.1152/jn.1994.71.1.375

[41] De Schutter E, Bower JM (1994) An active membrane model of the cerebellar purkinje cell ii. simulation of synaptic responses. Journal of Neurophysiology 71: 401–419. 8158238 10.1152/jn.1994.71.1.401

[42] Schultheiss NW, Edgerton JR, Jaeger D (2010) Phase response curve analysis of a full morphological globus pallidus neuron model reveals distinct perisomatic and dendritic modes of synaptic integration. The Journal of neuroscience: the official journal of the Society for Neuroscience 30: 2767–2782. 10.1523/JNEUROSCI.3959-09.2010 20164360 PMC2833015

[43] Stiefel KM, Gutkin BS, Sejnowski TJ (2008) The effects of cholinergic neuromodulation on neuronal phase-response curves of modeled cortical neurons. Journal of computational neuroscience 26: 289–301. 10.1007/s10827-008-0111-9 18784991 PMC2857973

[44] Miranda-Dominguez O, Netoff TI (2013) Parameterized phase response curves for characterizing neuronal behaviors under transient conditions. Journal of Neurophysiology 109: 2306–2316. 10.1152/jn.00942.2012 23365188

[45] Polhamus DG, Wilson CJ, Paladini CA (2012) Prc estimation with varying width intervals In: Phase Response Curves in Neuroscience, Springer pp. 163–177.

[46] Wilson CJ, Barraza D, Troyer T, Farries MA (2014) Predicting the responses of repetitively firing neurons to current noise. PLoS computational biology 10: e1003612 10.1371/journal.pcbi.1003612 24809636 PMC4014400

[47] Fujita T, Fukai T, Kitano K (2012) Influences of membrane properties on phase response curve and synchronization stability in a model globus pallidus neuron. Journal of computational neuroscience. 10.1007/s10827-011-0368-2 21993572

[48] Farries Ma, Wilson CJ (2012) Biophysical basis of the phase response curve of subthalamic neurons with generalization to other cell types. Journal of neurophysiology 108: 1838–55. 10.1152/jn.00054.2012 22786959 PMC3774581

[49] Akemann W, Knöpfel T (2006) Interaction of kv3 potassium channels and resurgent sodium current influences the rate of spontaneous firing of purkinje neurons. The Journal of neuroscience: the official journal of the Society for Neuroscience 26: 4602–12. 10.1523/JNEUROSCI.5204-05.2006 16641240 PMC6674064

[50] Tateno TT, Robinson HPC (2007) Phase resetting curves and oscillatory stability in interneurons of rat somatosensory cortex. Biophysical Journal 92: 683–695. 10.1529/biophysj.106.088021 17192317 PMC1751383

[51] Stigen T, Danzl P, Moehlis J, Netoff T (2009) Linear control of neuronal spike timing using phase response curves. Conference proceedings: Annual International Conference of the IEEE Engineering in Medicine and Biology Society IEEE Engineering in Medicine and Biology Society Conference 2009: 1541–1544. 10.1109/IEMBS.2009.5333079 19963758

[52] Ratté S, Hong S, De Schutter E, Prescott Sa (2013) Impact of neuronal properties on network coding: roles of spike initiation dynamics and robust synchrony transfer. Neuron 78: 758–72. 10.1016/j.neuron.2013.05.030 23764282 PMC3753823

[53] Hong S, Negrello M, Junker M, Smilgin A, Thier P, et al. Multiplexed coding by cerebellar purkinje neurons. Society For Neuroscience Annual Meeting 2014, number 632.16/JJ19. 10.7554/eLife.13810 PMC4961467 27458803

[54] Pasalar S, Roitman aV, Durfee WK, Ebner TJ (2006) Force field effects on cerebellar purkinje cell discharge with implications for internal models. Nature neuroscience 9: 1404–11. 10.1038/nn1783 17028585

[55] Stone L, Lisberger S (1990) Visual responses of purkinje cells in the cerebellar flocculus during smooth-pursuit eye movements in monkeys. i. simple spikes. Journal of Neurophysiology 63: 1241–61. 2358872 10.1152/jn.1990.63.5.1241

